# Spirulina supplementation improves oxygen uptake in arm cycling exercise

**DOI:** 10.1007/s00421-020-04487-2

**Published:** 2020-09-05

**Authors:** Tom Gurney, Owen Spendiff

**Affiliations:** School of Life Sciences, Kingston University, London, KT1 2EE UK

**Keywords:** Heart rate, Hemoglobin, Algae, RER

## Abstract

**Purpose:**

Spirulina has previously been reported to improve high-intensity exercise performance and hemoglobin. However, spirulina’s effect on arm cycling exercise has yet to be investigated. The purpose of this study was to investigate the responses of spirulina supplementation on hemoglobin and on oxygen uptake, RER and HR during seated arm cycling exercise.

**Methods:**

In a double-blinded randomized crossover design, eleven males untrained in arm cycling ingested 6 g/day of spirulina or placebo for seven days. Seated on the Arm Crank Ergometer, each participant performed a baseline V̇O_2max_ test, and then after supplementation, 2 × 30-min submaximal exercise bouts corresponding to 55% of their V̇O_2max_, followed by an incremental test to fatigue. A seven-day wash-out period was required between conditions. Oxygen uptake, RER and HR were measured continuously during exercise and hemoglobin measured prior to exercise after both conditions.

**Results:**

Spirulina significantly (*p* < 0.05) increased Hb in comparison to Placebo (144.1 g/l ± 10.5 Vs 154.5 g/l ± 6.9). After spirulina supplementation, during the 30-min exercise bouts, oxygen uptake and HR were significantly lower (2170 ml/min ± 173 Vs 2311 ml/min ± 189 and 154 bpm ± 14 Vs 149 bpm ± 17), RER was not significantly different. In comparison to placebo, Spirulina significantly increased oxygen uptake at time of fatigue (34.10 ml/min/kg ± 6.03 Vs 37.37 ml/min/kg ± 5.98). Time taken to fatigue was not different.

**Conclusion:**

Spirulina supplementation significantly reduces oxygen uptake and HR during arm cycling submaximal exercise, allowing for an increased oxygen uptake during an incremental test to fatigue.

## Introduction

Global demand for algae is continuously increasing and it has been reported that algae is now being consumed beyond the traditional benefits for nutrition and health (Wells et al. 2016). The exploration and interest of algae as a ‘functional food’ is evident from recent reviews whereby a variety of papers have reported potential positive outcomes from supplementation (Dominguez 2013; Levine and Fleurence 2018; Wells et al. 2016; Wu et al. 2016). Spirulina (SP) is known for its multicomponent properties which include high levels of micronutrients, proteins, vitamins and minerals (Kalafati et al. 2010). Initial investigations using SP have predominantly been within a clinical health remit, such as improving blood morphological parameters and immune function (Kelkar et al. 2008; Milasius et al. 2009; Selmi et al. 2011). However recently, there has been more focus on SP’s antioxidant potential when comparing exercise-induced oxidative stress markers (Lu et al. 2006; Kalafati et al. 2010; Kalpana et al. 2017; Hernández-Lepe et al. 2018; Franca 2010), with the consensus attributing the constituents of SP to the activation of cellular antioxidant enzymes, inhibition of lipid peroxidation and free radicals, whilst also increasing the activity of superoxide dismutase (Wu et al. 2016).

The ergogenic aid capabilities of SP have previously been explored in running and cycling whereby increases in time to fatigue and/or exhaustion were reported (Lu et al. 2006; Kalafati et al. 2010; Hernández-Lepe et al. 2018). Specifically, Kalafati et al. (2010) observed a significant decrease in carbohydrate oxidation and significant increases in fat oxidation during a 2-h run at 70–75% V̇O_2max_. It was suggested that these changes in substrate oxidation may have consequently led to a sparing of glycogen stores, which therefore may have facilitated high-intensity exercise to continue for a longer period of time. However, considering previous research has demonstrated positive outcomes in blood morphological parameters, such as Hb, after SP supplementation (Kelkar et al. 2008; Milasius et al. 2009; Selmi et al. 2011); thus far, researchers have failed to consider that these small increases in Hb from SP may also be the ergogenic aid for athletes.

An in vitro Digestion/Caco-2 Cell Culture Model investigation reported that the iron found in SP has a high bioavailability (Puyfoulhoux et al. 2001) and Buratti et al. (2015) described iron as an essential nutrient for endurance athletes, indispensable for hemoglobin (Hb) production in the blood. Indeed, it is well established that Hb is essential for the transportation of oxygen from the lungs to the working skeletal muscles (Hinton 2014; Mairbäurl 2013; Otto et al. 2013) and even small increases in Hb are associated with improved oxygen uptake during exercise (Mairbäurl 2013). For instance, linear regression analysis has demonstrated that for every 3 g/L increase in Hb concentration V̇O_2max_ could be increased by ~ 1% (Mairbäurl 2013). As such, any small changes in Hb derived from the high iron content in SP may act as an ergogenic aid for athletes.

To date, the handful of studies that have directly investigated the ergogenic aid capabilities of SP have only examined lower body modality exercise (Franca et al. 2010; Hernández-Lepe et al. 2018; Kalafati et al. 2010; Kalpana et al. 2017; Lu et al. 2006). Upper body sports, such as kayaking, canoeing, and wheelchair events, also require athletes to exert themselves with regular submaximal and high-intensity interval exercise bouts during training and competition. Importantly, the hemodynamics of upper body exercise are different when compared to lower body exercise (Sawka 1986). For example, arm cycling exercise utilizes a smaller active muscle mass with a reduced oxidative capacity resulting in a greater and/or earlier recruitment of type II muscle fibers (Koppo et al. 2002). These muscle fibers can be more prone to fatigue and produce deleterious by-products due to their reliance on anaerobic glycolysis (Koga et al. 1996; Koppo et al. 2002). However, blood flow per unit muscle mass is higher during arm cycling exercise than during leg cycling exercise at the same relative VO_2_ (Koppo et al. 2002). Considering that previous research has highlighted the effectiveness of SP on blood morphological parameters (Kelkar et al. 2008; Milasius et al. 2009; Selmi et al. 2011), the positive changes could be more influential for athletes using only their upper body for exercise. Therefore, the purpose of this study was to investigate whether a one-week supplementation period using 6 g/day dosage of SP could elicit an ergogenic effect on upper body cycling exercise.

## Methodology

### Study design

A double-blind randomized cross-over design was employed to examine the influences of SP supplementation on blood Hb and respiratory variables, during a 30-min bout of submaximal upper body cycling exercise at 55% V̇O_2max_, followed by an incremental test to fatigue using Arm Crank Ergometry (ACE). Oxygen uptake (ml/min), Respiratory Exchange Ratio (RER) and Heart rate (bpm) were compared across both conditions during submaximal exercise bouts. Hemoglobin was compared after each supplementation period. Additionally, time taken to fatigue (seconds) and oxygen uptake (ml/kg/min) at the time of fatigue were compared across both supplement conditions.

Participants were required to visit the laboratory on four separate occasions in a 4-h post-prandial fasted state. Participants were also asked to refrain from exercise 48 h before each visit. The first visit comprised baseline anthropometric measurements and a V̇O_2max_ test. On the second visit, participants were required to attend the laboratory to accustom them to the arm cycling protocol prior to the supplementation conditions. Participants were then randomly allocated to either SP or soy protein (placebo) capsules and were instructed to ingest 6 g each day (14 capsules: 5 with breakfast, 5 with lunch, 4 with dinner) for 7 days. All capsules were visually identical, and there were no reports of any taste differences nor gastrointestinal issues during/after each supplementation period. Capsules were placed into 7 small paper day bags and were coded by an independent lab technician. Between the third and fourth visit to the laboratory, there was a minimum of 14 days respite to allow for a full 7-day wash-out period and the subsequent 7-day supplementation period. Throughout the supplementation process, participants were asked to refrain from taking any additional vitamin products and 24 h prior to testing, participants were asked not to consume alcohol or complete any strenuous exercise.

### Participants

Eleven healthy males, unfamiliar with arm cycling exercise, were recruited to participate in the present study (Mean ± SD; Age 21 ± 1 years, Stature 182.3 ± 8.9 cm, Mass 77.5 ± 17.2 kg). Each participant was provided information outlining the tests and required to provide their written informed consent prior to any testing. The Faculty of Science, Engineering and Computing Ethics Committee at Kingston University London approved the study in accordance to the Declaration of Helsinki. Any volunteer that currently smoked or had a history of cardiovascular disease was excluded.

### Baseline measurements and ($\dot{\mathrm{V}}{\mathrm{O}}_{2\mathrm{max}}$)

Controlling for the exact same time of day, each participant came into the laboratory, each visit consisted of basic anthropometric measurements of stature (cm) (Floor Stadiometer, Holtain Ltd., Dyfed, Wales) and mass (kg) (Bodystat 1500, Bodystat Ltd., Isle of Man, UK) and thereafter, a small fingertip blood sample was taken from each participant whereby it was placed into the HemoCue Hb 2001 + (HemoCue AB, Ängelholm, Sweden) for hemoglobin analysis.

Using a Tape Measure (Bodycare Products Ltd., Northfield Road, Southam, Warwickshire, UK), the desired comfortable arm crank height for each participant was recorded by measuring the distance between the top of the ergometer and the top of the middle bracket which held the ergometer in place and this height was replicated on each visit. Additionally, each participant was instructed to adjust the seating position until a comfortable distance from the arm crank ergometer was achieved and this was also recorded for each subsequent visit.

The Oxycon Pro mask (VIASYS GmbH, Eric Jaeger, Hoechberg, Germany) was placed onto the participant comfortably whereby respiratory variables were measured throughout the testing. Heart rate was recorded (Polar Electro Oy, Kempele, Finland) after the warm-up and continuously at every minute until volitional fatigue. A V̇O_2max_ ramp incremental protocol (Smith et al. 2007) was conducted on the Arm Crank Ergometer (Angio cpet 967904). The test comprised an initial 2-min warm-up period (resistance 50 W) at 70RPM. Thereafter, the intensity increased 20 W every 2 min. The respiratory variables were analyzed and averaged at every 15 s time frame. The V̇O_2max_ was determined by the highest VO_2_ value that was recorded from the 15 s averages and WRmax was rounded down to the nearest incremental stage. The maximum V̇O_2max_ and WRmax score were recorded to subsequently establish each participant 55% relative intensity for the 30-min submaximal exercise sessions.

### Submaximal test and incremental test to fatigue

The corresponding resistance for each participant’s 55% relative intensity work rate was applied onto the ergometer. The ergometer crank height and seat were manually adjusted in accordance to their position from the baseline visit. The Oxycon Pro mask and HR strap were fitted as before and thereafter participants were instructed to maintain their RPM between 60 and 70. To reduce any noise from anticipatory rise effects, the first 5 min of values from oxygen uptake, RER and HR during the submaximal exercise tests were not recorded. At every 5-min interval, HR was recorded. Oxygen uptake and RER were averaged to 15 s whereby each 5-min interval was then calculated and averaged for analysis. Following the submaximal exercise test, participants were given a 5-min rest period, when thereafter an incremental test to fatigue was conducted using the exact same protocol from the V̇O_2max_ test. At the point of fatigue, time taken (seconds) and oxygen uptake (ml/min/kg) were recorded for comparison between conditions.

### Statistics

Data are presented as mean ± SD. All statistical procedures were carried out using IBM SPSS version 24 for windows. All datasets were analyzed for normality using Shapiro–Wilk, while Mauchly’s test of Sphericity was employed to establish any potential violations. Statistical significance for alpha was set at 0.05. In addition, effect size (calculated using Partial ETA squared), observed power and confidence intervals were used when appropriate. Oxygen uptake, RER and HR were analyzed using a two-way within subjects repeated measures ANOVA with a Bonferroni correction for multiple comparisons to determine any differences during the submaximal exercise. Any violations of sphericity were corrected using values from the Greenhouse–Geisser. Where significant main effects were identified, post hoc Paired Sample *T*-Tests were employed to determine any statistically significant differences within the data. Variables including Hb, oxygen uptake, and the time taken at the point of fatigue were also compared using a paired sample *T*-Test.

## Results

### Hemoglobin

A significant increase in Hb from placebo was observed following the consumption of SP (*P* < 0.05), see Table 1.

**Table 1:** Average hemoglobin (Mean and SD) values following the supplementation period

	Placebo	Spirulina	*P* value
Hemoglobin (g/L)	144.1 ± 10.5	154.5 ± 6.9	0.005

### Submaximal testing

#### Oxygen uptake

**Fig. 1:**
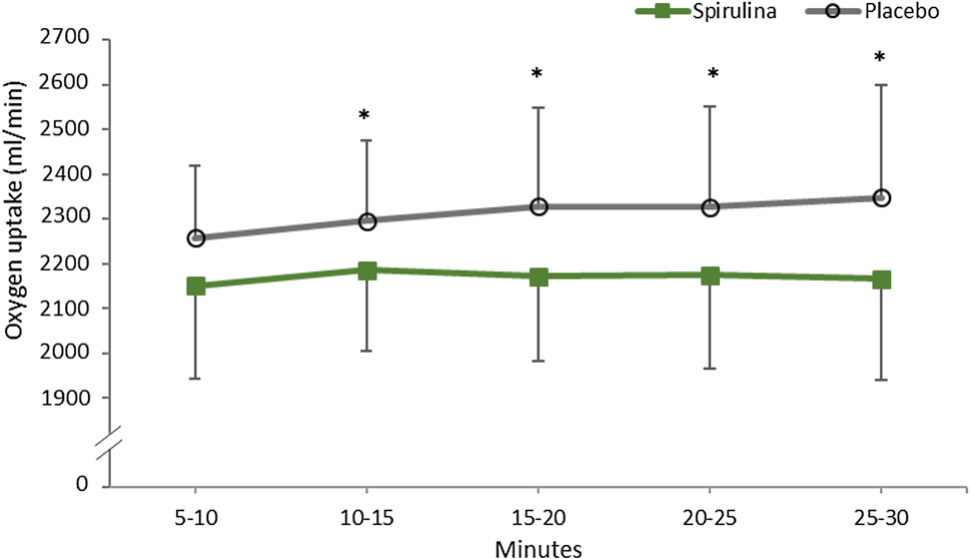
Oxygen uptake (ml/min) during the 30-min submaximal exercise bout following the 7-day supplementation of Spirulina or Placebo. *Signifies *P* <0 .05

During the 30-min steady state submaximal exercise tests, participants elicited a significantly lower total average oxygen uptake (*P* = 0.03, ETA = 0.389, Observed Power = 0.625) after the supplementation of SP (2169.9 ± 202.5 ml/min) in comparison to Placebo (2310.8 ± 207.9). Post hoc tests revealed oxygen uptake to be significantly lower between conditions after 10 min and remained so until the completion of the test (Fig. 1). No significant main effect or interaction between oxygen uptake and time or supplement and time was observed (*P* > 0.05).

#### Heart rate

**Fig. 2:**
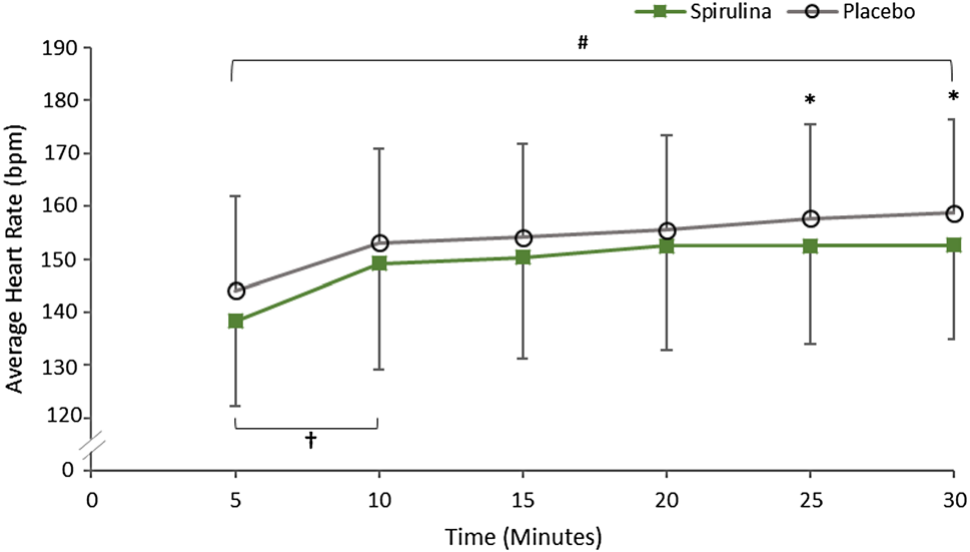
Heart rate (bpm) during the 30-min submaximal exercise bout following the 7-day supplementation of Spirulina or Placebo. *Signifies a significant difference between supplements *P* < 0.05, #signifies a significant within-trial increase in HR across every 5-min interval in the Placebo condition *P* <0 .05, †signifies a significant within-trial increase in HR across the 5th–10th minute in the Spirulina condition *P* < 0.05

Total average HR was significantly lower between trials during the 30-min submaximal exercise bout (*P* = 0.022, ETA = 0.423, Observed Power = 0.685) following the supplementation of SP (149 ± 18 bpm) in comparison to Placebo (154 ± 14 bpm). Post hoc Paired Sample T-Tests exhibited statistical significance between the 25th min (*P* = 0.006, 95% CI − 10.04 to − 2.13) and the 30th min (*P* = 0.017, 95% CI − 9.05 to − 1.12) (Fig. 2). A significant within-trial increase in HR was observed for both conditions (*P* < 0.05). Heart rate over time in the SP condition showed a significant incline at the first 5 min followed by a plateau from the 10th min onwards. Heart rate over time in the Placebo condition significantly increased every 5 min over the duration of the trial. There was no interaction for supplement and time (*P* > 0.05).

#### Incremental test to fatigue

After the supplementation of SP, there was an 8.9% increase in oxygen uptake at the point of fatigue, revealing a statistical increase being met between SP (37.37 ± 5.98 ml/kg/min) and placebo (34.10 ± 6.03 ml/kg/min, *P* = 0.024, 95% CI − 0.51 to 6.02). Average time to fatigue in the SP condition was 530 ± 68 s in comparison to 503 ± 79 s in the placebo condition, no statistical difference was met (*P* = 0.113).

### RER

Respiratory Exchange Ratio for SP (1.00 ± 0.06) was not different (*P* = 0.874) to placebo (1.01 ± 0.07). Within-trial analysis demonstrated a decline in RER in both conditions from 0–20 min (*P* < 0.05), which followed a plateau in RER whereby no significant difference was observed. There was no interaction for supplement and time (*P* > 0.05).

## Discussion

**Fig. 3:**
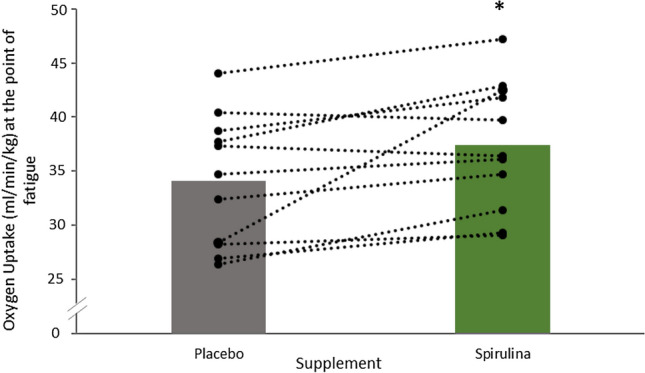
Average and individual oxygen uptake of participants at the point of fatigue following an incremental test after the 7-day supplementation of Spirulina or Placebo. *Signifies a statistically significant increase after Spirulina supplementation *P* < 0.05

To date, this appears to be the first study to investigate the ergogenic capability of SP and its relationship for improving Hb whilst comparing key respiratory variables during arm cycling exercise. The novel findings of this study were that 6 g a day supplementation of SP for seven days significantly reduced oxygen uptake (Fig. 1) and heart rate (Fig. 2) during arm cycling submaximal exercise bouts. Spirulina further elicited a significant increase in Hb (Table 1) and increased oxygen uptake during an incremental test to fatigue (Fig. 3).

### Hemoglobin

Results from the present study are consistent with previous literature whereby the supplementation of SP elicited significant increases in Hb (Milasius et al. 2009; Selmi et al. 2011; Uliyar et al. 2000). Given the consistency of results with positive changes in Hb after the supplementation of SP, it can also be assumed that compliance during the study was good. Previously low daily doses and long intervention periods of 2.25 g for 14 days (Milasius et al. 2009), 3 g for 12 weeks (Selmi et al. 2011) and 5 g for 30 days (Uliyar et al. 2000) were considered effective for increasing Hb following the supplementation of SP. The shorter intervention period of seven days with a higher daily dose of 6 g/day employed in this study also generated a significant increase in Hb (Table 1), further demonstrating the efficacy in the assimilation of iron from SP (Milasius et al. 2009). Mechanistically, it has been suggested that this high absorption of iron may occur due to the absence of phytate and oxalate in algae (García-Casal et al. 2007; Gutiérrez-Salmeán et al. 2015), both of which have previously been reported to inhibit iron absorption due to binding and forming insoluble complexes with iron (Walter 1997). Indeed, iron plays a fundamental role in daily Hb synthesis and given the importance of possessing optimal iron and Hb values for endurance performance (Hinton 2014; Mairbäurl 2013; Otto et al. 2013), increases in both from the consumption of SP could, therefore, make it a highly attractive supplement.

### Submaximal tests

**Fig. 4:**
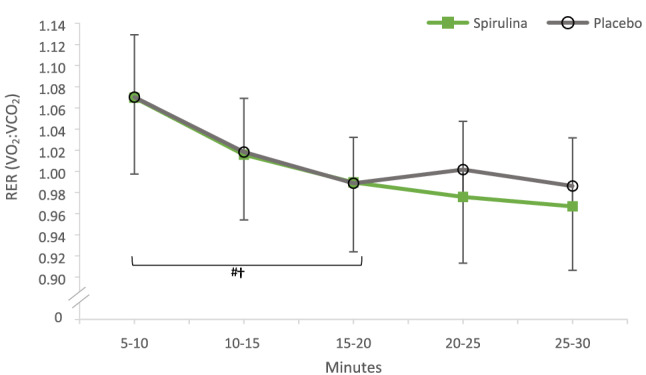
RER (VO_2_:VCO_2_) during the 30-min submaximal exercise bout following the 7-day supplementation of Spirulina or Placebo. #†signifies a significant within-trial decrease in RER across the first 20 min in both the Spirulina (†) and Placebo (#) conditions (*P* < 0.05)

The significant reduction in oxygen uptake (Fig. 1) and HR (Fig. 2) during submaximal intensity gives a first insight into SP’s ergogenic aid capabilities during arm cycling exercise. This capacity to perform arm cycling submaximal exercise bouts with reduced cardiovascular and respiratory demand could be attributed to several mechanisms of action. Although the exact mechanism behind these physiological changes is difficult to ascertain, a plausible explanation behind a lower oxygen uptake may be associated with the 7.9% increase in Hb from SP supplementation. One of the Hb’s key roles is the transportation of oxygen from the lungs to the working skeletal muscles (Hinton 2014; Mairbäurl 2013; Otto et al. 2013). Moreover, when ATP demand is high in the working muscles during exercise, a decrease in the Hb–O_2_ affinity favors the release of oxygen from the Hb molecule to support oxidative phosphorylation (Mairbäurl 2013). Hence, increases in Hb have been associated with enhancing aerobic oxidative capacity during exercise (Otto et al. 2013). Therefore, in the present study, the 7.9% increase in Hb may have resulted in a lower oxygen uptake and HR during the submaximal exercise bouts. This enhanced oxidative capacity is further supported by a lowering RER trend in the SP condition from the 20th min onwards (Fig. 4). Indicating that during the last 10 min, participants began marginally oxidizing and metabolizing fat at a higher rate. Mechanistically, Kalafati et al. (2010) previously suggested that an increase in circulatory linolenic acid from SP may have altered fat metabolism, further research is needed to confirm this. Despite RER not reaching a significant difference, this may have potentiated the preservation of glycogen stores and reduced the reliance of carbohydrate oxidation for energy, consistent with previous literature (Kalafati et al. 2010).

Post hoc tests within the Placebo condition demonstrated a significant increase in HR from the 5th min and at every 5-min interval thereafter (see Results—Heart rate), displaying a continuous increase over time. This coupled with marginally higher RER values in the placebo condition during the last 10 min seems to be consistent with the apparent trend in the present study whereby participants found the last 10 min particularly difficult (Fig. 2). Arm cycling submaximal exercise bouts in the placebo condition perhaps resulted in a higher reliance on anaerobic fast twitch muscle fibers. Smaller skeletal type II muscles fibers are predominantly used during ACE which are typically untrained, possess fewer mitochondria and, therefore, have the tendency to increase deleterious waste products within the blood and muscle (Koppo et al. 2002). This increases the possibility of an increasing rate of metabolic acidosis and oxidative stress. To redress this metabolic unbalance, increases in HR and oxygen demand to the working muscles occur, as can be seen in Figs. 1, 2. Indeed, this apparent higher reliance on fast twitch muscles fibers may have also resulted in a higher demand in carbohydrate oxidation during the last 10 min (Fig. 4).

Conversely, the supplementation of SP significantly lowered the average HR between conditions. Kalpana et al. (2017) reported similar findings when comparing HR recovery values post cardiopulmonary exercise testing on a bike. Post hoc analysis in the SP condition revealed there to be a plateau in HR from the 10th min onwards. A speculated physiological mechanism of action for a significantly lower HR is that SP contains arginine (Lafarga et al. 2020). Arginine is an essential amino acid associated with augmenting the bioavailability of Nitric Oxide (NO), a well-established signaling molecule associated with endothelium vasodilation (Hishkawa et al. 1992). Similarly, the phycocyanin constituent of SP has previously been reported to increase the expression of Endothelial Nitric Oxide Synthase (eNOS) in rats (Ichimura et al. 2013). This localized vasodilation from NO increases blood flow which may improve peripheral oxygen offload to working muscles and efflux of deleterious by-products. Although further research is required in humans, it could be suggested that a similar mechanism of action may be occurring in the present study. Synergistically, the increased circulatory Hb, phycocyanin and arginine from SP can present complementary and overlapping mechanisms of action for improving oxygen uptake. As such, in the present study, this may have resulted in a lower steady state oxygen uptake (Fig. 1) and lower HR (Fig. 2) at a given submaximal intensity, whilst also demonstrating a marginal increasing trend in fat oxidation (Fig. 4).

### Incremental test to fatigue

The results from the current study further support the notion and are consistent with previous literature whereby SP can improve high-intensity exercise (Hernández-Lepe et al. 2018; Kalafati et al. 2010; Kalpana et al. 2017; Lu et al. 2006). However, the aforementioned studies attributed their findings to SP’s antioxidant capabilities, such as increasing the scavenging of reactive oxygen nitrogen species and reducing oxidative stress biomarkers (Malondialdehyde and Creatine Kinase). It cannot be ignored that the protective antioxidant capabilities of SP might also have had some influence on oxygen uptake during the incremental test, however, measurement of these was beyond the scope of the present study.

Mechanistically, the multicomponent SP species could have several other physiological mechanisms of action for improving high-intensity exercise. First, it is well established from early seminal work that Hb is correlated with incremental exercise tests (Bassett and Howley 2000; Ekblom et al. 1972; Kjellberg et al. 1949; Schmidt and Prommer 2010), and that oxygen delivery is a primary limitation during maximal exertion (Bassett and Howley 2000; Goodrich et al. 2018). Additionally, during high-intensity exercise, Hb fulfills an important buffering role by maintaining blood pH via the transport of CO_2_, lactate, and by the binding of H + to Hb (Mairbäurl 2013), this function is particularly important during the later stages of an incremental test. The 7.9% increase in Hb in the present study could, therefore, possibly explain the 8.9% increase in oxygen uptake at the point of fatigue. Indeed, Hb concentration increased by 10.4 g/L, which according to Otto et al. (2013) could at least equate for 3% of the 8.9% improvement in oxygen uptake observed in this study.

Another possible explanation as to why there was an 8.9% increase in oxygen uptake at the time of fatigue could possibly be owed to a marginal reduction in RER during the last 10 min in the submaximal exercise bouts (Fig. 4). This potential increase in fat oxidation and reduction in carbohydrate oxidation may have allowed for a greater preservation of glycogen or glucose stores just before the incremental test to fatigue (Kalafit et al. 2010). Subsequently, during the incremental test, this may have allowed participants to continue exercising for longer (despite not reaching statistical significance) and increase their oxygen uptake at the point of fatigue. On the other hand, in the placebo condition, an earlier increase in metabolic acidosis and carbohydrate oxidation may have reduced the oxidative capacity in the latter stages of the incremental test, resulting in an earlier onset of fatigue.

Finally, the arginine content within SP may have also played a key role at increasing oxygen uptake during an incremental test to fatigue. Arginine plays a key role in endothelium vasodilation whereby it augments the bioavailbity of NO (Hishkawa et al. 1992). This vasodilation can consequently increase blood flow to the working muscles which has previously been reported to improve time to fatigue (Álvares et al. 2011).

One key limitation of this study was that all participants from this study were only male individuals. Given the prevalence of sports anemia, particularly in the female population (Hinton 2014; Parks et al. 2017), SP may be especially useful to upper body endurance female athletes.

## Practical application and conclusion

In conclusion, seven days’ SP supplementation of just 6 g per day significantly increased Hb whilst also reducing oxygen uptake and HR during arm cycling submaximal exercise. This subsequently allowed for an increased oxygen uptake during an incremental test to fatigue. Collectively, these results contribute to the relatively small body of research whereby SP can be considered an effective ergogenic aid for both submaximal and maximal intensities and particularly for arm cycling exercise modalities where oxygen uptake can be a limiting factor. Given the shorter supplementation period and higher dosage used in this study compared to previous research, the positive findings of this study illustrate that the optimum dosage of SP and precise mechanisms for enhancing performance are still to be elucidated; however, these findings are adding to the body of knowledge on a novel supplement.

## Abbreviations

ACE: Arm Crank Ergometry
ATP: Adenosine Triphosphate
CO_2_: Carbon Dioxide
eNOS: Endothelial Nitric Oxide Synthase
Hb: Hemoglobin
HR: Heart rate
NO: Nitric Oxide
RBC: Red Blood Cells
RER: Respiratory Exchange Ratio
RPM: Revolutions Per Minute
SP: Spirulina

## References

[CR1] Álvares TS, Meirelles CM, Bhambhani YN (2011). L-arginine as a potential ergogenic aid in healthy subjects. Sports Med.

[CR2] Bassett DR, Howley ET (2000). Limiting factors for maximum oxygen uptake and determinants of endurance performance. Med Sci Sports Exerc.

[CR3] Buratti P, Gammella E, Rybinska I, Cairo G, Recalcati S (2015). Recent advances in iron metabolism. Med Sci Sports Exerc.

[CR4] Dominguez H (2013). Functional ingredients from algae for foods and nutraceuticals.

[CR5] Ekblom B, Goldbarg AN, Gullbring B (1972). Response to exercise after blood loss and reinfusion. J Appl Physiol.

[CR6] Franca G, Silva A, Costa M (2010). Spirulina does not decrease muscle damage nor oxidative stress in cycling athletes with adequate nutritional status. Biol Sport.

[CR7] García-Casal M, Pereira A, Leets I, Ramírez J, Quiroga M (2007). High iron content and bioavailability in humans from four species of marine algae. J Nutr.

[CR8] Goodrich J, Ryan B, Byrnes W (2018). The influence of oxygen saturation on the relationship between hemoglobin mass and VO2max. Sports Med Int Open.

[CR9] Gutiérrez-Salmeán G, Fabila-Castillo L, Chamorro-Cevallos G (2015). Nutritional and toxicological aspects of spirulina (Arthrospira). Nutr Hosp.

[CR10] Hernández-Lepe M, López-Díaz J, Juárez-Oropeza M, Hernández-Torres R, Wall-Medrano A, Ramos-Jiménez A (2018). Effect of *Arthrospira* (Spirulina) maxima supplementation and a systematic physical exercise program on the body composition and cardiorespiratory fitness of overweight or obese subjects: a double-blind, randomized, and crossover controlled trial. Mar Drugs.

[CR11] Hinton P (2014). Iron and the endurance athlete. Appl Physiol Nutr Metab.

[CR12] Hishikawa K, Nakaki T, Tsuda M (1992). Effect of systemic l-arginine administration on hemodynamics and nitric oxide release in man. Jpn Heart J.

[CR13] Ichimura M, Kato S, Tsuneyama K (2013). Phycocyanin prevents hypertension and low serum adiponectin level in a rat model of metabolic syndrome. Nutr Res.

[CR14] Kalafati M, Jamurtas A, Nikolaidis M (2010). Ergogenic and antioxidant effects of spirulina supplementation in humans. Med Sci Sports Exerc.

[CR15] Kalpana K, Kusuma D, Lal P, Khanna G (2017). Impact of spirulina on exercise induced oxidative stress and post exercise recovery heart rate of athletes in comparison to a commercial antioxidant. Food Nutr J.

[CR16] Kelkar G, Subhadra K, Chengappa R (2008). Effect of antioxidant supplementation on hematological parameters, oxidative stress and performance of Indian athletes. J Hum Ecol.

[CR17] Kjellberg SR, Rudhe U, Sjöstrand T (1949). The amount of hemoglobin (blood volume) in relation to the pulse rate and heart volume during work. Acta Physiol Scand.

[CR18] Koga S, Shiojiri T, Shibasaki M, Fukuba Y, Fukuoka Y, Kondo N (1996). Kinetics of oxygen uptake and cardiac output at onset of arm exercise. Respir Physiol.

[CR19] Koppo K, Bouckaert J, Jones A (2002). Oxygen uptake kinetics during high-intensity arm and leg exercise. Respir Physiol Neurobiol.

[CR20] Lafarga T, María Fernández-Sevilla J, González-López C, Gabriel Acién-Fernández F (2020). Spirulina for the food and functional food industries. Food Res Int.

[CR21] Levine I, Fleurence J (2018). Microalgae in health and disease prevention.

[CR22] Lu H, Hsieh C, Hsu J, Yang Y, Chou H (2006). Preventive effects of *Spirulina* platensis on skeletal muscle damage under exercise-induced oxidative stress. Eur J Appl Physiol.

[CR23] Mairbäurl H (2013). Red blood cells in sports: effects of exercise and training on oxygen supply by red blood cells. Front Physiol.

[CR24] Milasius K, Malickaite R, Dadeliene R (2009). Effect of spirulina food supplement on blood morphological parameters, biochemical composition and on the immune function of sportsmen. Biol Sport.

[CR25] Otto J, Montgomery H, Richards T (2013). Haemoglobin concentration and mass as determinants of exercise performance and of surgical outcome. Extrem Physiol Med.

[CR26] Parks R, Hetzel S, Brooks M (2017). Iron deficiency and anemia among collegiate athletes. Med Sci Sports Exerc.

[CR27] Puyfoulhoux G, Rouanet J, Besançon P, Baroux B, Baccou J, Caporiccio B (2001). Iron availability from iron-fortified spirulina by an in vitro digestion/caco-2 cell culture model. J Agric Food Chem.

[CR28] Sawka M (1986). Physiology of upper body exercise. Am J Sports Med.

[CR29] Schmidt W, Prommer N (2010). Impact of alterations in total hemoglobin mass on VO2max. Exerc Sport Sci Rev.

[CR30] Selmi C, Leung P, Fischer L (2011). The effects of *Spirulina* on anemia and immune function in senior citizens. Cell Mol Immunol.

[CR31] Smith P, Doherty M, Price MJ (2007). The effect of crank rate strategy on peak aerobic power and peak physiological responses during arm crank ergometry. J Sports Sci.

[CR32] Uliyar M, Alefia S, Uma I, Panam P (2000). The effect of *Spirulina* supplementation on blood haemoglobin levels of anaemic adult girls. J Food Technol.

[CR33] Walter P (1997). Effects of vegetarian diets on aging and longevity. Nutr Rev.

[CR34] Wells M, Potin P, Craigie J (2016). Algae as nutritional and functional food sources: revisiting our understanding. J Appl Psychol.

[CR35] Wu Q, Liu L, Miron A, Klímová B, Wan D, Kuča K (2016). The antioxidant, immunomodulatory, and anti-inflammatory activities of Spirulina: an overview. Arch Toxicol.

